# Baseline Susceptibility of *Culiseta longiareolata* (Diptera: Culicidae) to Different Imagicides, in Eastern Azerbaijan, Iran

**Published:** 2019-12-31

**Authors:** Teimour Hazratian, Azim Paksa, Mohammad Mahdi Sedaghat, Hassan Vatandoost, Seyed Hassan Moosa-Kazemi, Alireza Sanei-Dehkordi, Yaser Salim-Abadi, Masoumeh Pirmohammadi, Saideh Yousefi, Masoumeh Amin, Mohammad Ali Oshaghi

**Affiliations:** 1 Departmemt of Parasitology, Faculty of Medicine, Tabriz University of Medical Sciences, Tabriz, Iran; 2 Department of Medical Entomology and Vector Control, School of Public Health, Tehran University of Medical Sciences, Tehran, Iran; 3 Department of Environmental Chemical Pollutants and Pesticides, Institute for Environmental Research, Tehran University of Medical Sciences, Tehran, Iran; 4 Department of Medical Entomology and Vector Control, Faculty of Health, Hormozgan University of Medical Sciences, Bandar Abbas, Iran; 5 Infectious and Tropical Diseases Research Center, Hormozgan Health Institute, Hormozgan University of Medical Sciences, Bandar Abbas, Iran; 6 Department of Health Services and Health Promotion, School of Health, Rafsanjan University of Medical Sciences, Rafsanjan, Iran

**Keywords:** Baseline susceptibility, *Culiseta longiareolata*, Insecticides

## Abstract

**Background:**

*Culiseta longiareolata* is an important vector for many human diseases such as brucellosis, avian influenza and West Nile encephalitis. It is likely an intermediate host of avian *Plasmodium* that can transmit Malta fever. The aim of this study was to determine the susceptibility level of *Cs. longiareolata* to different classes of imagicides which are recommended by World Health Organization .

**Methods:**

Larval stages of the *Cs. longiareolata* were collected from their natural habitats in Marand County at East Azerbaijan Province, northwestern of Iran in 2017. Adult susceptibility test were carried out with using impregnated papers to insecticides including DDT 4%, Cyfluthrin 0.15%, Deltamethrin 0.05%, Propoxur 0.1% and Fenitrothion 1% by standard test kits.

**Results:**

Results showed that *Cs. longiareolata* adult is more susceptible to pyrethroid and carbamate insecticides. Among tested insecticides, Cyfluthrin was the most toxic against *Cs. longiareolata* with LT_50_ value of 11.53 minutes and Fenitrothion had the least toxic effect (LT_50_: 63.39 min).

**Conclusions:**

This study provided a guideline for monitoring and evaluation of insecticide susceptibility tests against *Cs. longiareolata* mosquitoes for further decision making.

## Introduction

Mosquitoes transmit many important human diseases such as malaria, filariasis, several types of encephalitis, many arboviral diseases and also cause serious nuisance and irritation (1–5). West Nile virus has been detected in 62 mosquito species, including genera of *Aedes*, *Anopheles*, *Culiseta* and *Culex* in the United State of America (6–9). About 3500 species of mosquitoes reported worldwide, and approximately 64 of those can be found in Iran (10–17). *Culiseta longiareolata* is a vector for brucellosis, avian influenza and West Nile encephalitis. These mosquitoes are likely an intermediate host of avian *Plasmodium* and can transmit Malta fever (18, 19). The mosquito *Cs. longiareolata* is a common and abundant species in many countries of Europe, Africa and also Asia, such as Iran, Albania, Azores, Botswana, Bulgaria, Canary islands, Croatia, Cyprus, Djibouti, Egypt, southern England, Ethiopia, France, Greece, Hungary, India, Iraq, Italy, Jordan, Lebanon, Lesotho, Madeira, Mauritania, Morocco, Namibia, Pakistan, Portugal, Romania, Russia, Slovakia, Somalia, South Africa, Spain, Sudan, Switzerland, Syria, Tajikistan, Tunisia, Turkey, Ukraine and Yemen (1). Eggs and larvae of *Cs. Longiareolata* is found mostly in tires, so it can be spread across the world through tire trading (20). *Culiseta longiareolata* is found common in human habitations. The larvae are rarely found in natural waters that are found mostly in temporary pools, rock pools, artificial containers, wooden and metal barrels and tanks built of concrete, which are rich in decaying organic materials (18). Early growth stages larvae of *Cs. longiareolata* are more found in shallow areas of pools, whereas late growth stages are found deeper areas of the pools (21).

Chemical insecticides such as organophosphates, organochlorine, carbamate, and pyrethroid are principal weapon against both adult and larval stages of mosquitoes vectors (22–24). Increasing and inappropriate use of synthetic insecticides in mosquito control in parallel to pest control agriculture is one of the main causes of increased tolerance and resistance in different species of mosquitoes across the world (25–27). According to reports in recent years, the level of tolerance and resistance of some mosquitoes and other arthropods has increased in some parts of the world which is a major barrier to the success of vector control programs (28–34). In our knowledge, there was no comprehensive study on monitoring the susceptibility level of *Cs. longiareolata* to various insecticides in the world. The aim of this study was to evaluate the susceptibility of *Cs. longiareolata* to five common insecticides recommended by World Health Organization.

## Materials and Methods

### Study area

**Fig. 1.**
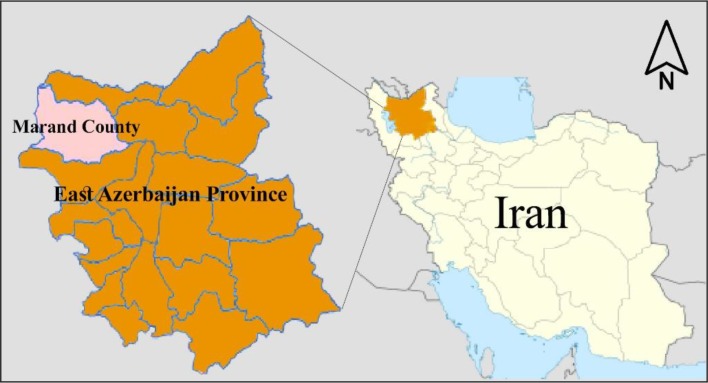
Map showing Iran, highlighting the location of East Azerbaijan Province and Marand County

This study was carried out in Marand County in East Azerbaijan Province, northwestern of Iran. The county located at latitude 38°42′N, longitude 45°76′E and altitude 1342 Meter (Fig. 1).

### Bioassay procedure

In this experimental study larval stage of *Cs. longiareolata* were collected from larval habitats, then all specimens were transferred to insectary of Department of Medical Entomology and Vector Control with 27±1 °C temperature, 12:12 light and dark period and 60±5% of relative humidity. Adult susceptibility test of mosquitoes were carried out using standard impregnated papers insecticides such as DDT 4%, Cyfluthrin 0.15%, Deltamethrin 0.05%, Propoxur 0.1% and Fenitrothion 1%. According to the standard procedures recommended by the World Health Organization (WHO). In brief, twenty-five unfed female mosquitoes were exposed to insecticide-impregnated papers at different exposure interval times, moreover for each different exposure time 4 replicates of mosquitos were used and 2 replicates of 25 adult mosquitoes were considered as controls with untreated papers.

Probit analysis was conducted on mortality data collected after 24 hours exposure to different times of insecticides using Finney’s statistical method to determine the lethal time causing for 50% and 90% mortality (LT_50_ and LT_90_) values and their 95% confidence limit of upper and lower confidence levels (35–37). The percentage mortality was calculated and corrections for mortality when necessary were done by Abbot’s formula (38). According to the WHO criteria, the susceptibility level of the mosqueitoes was considered in three classes as susceptible, tolerant and resistant. The mortality between 98–100% was considered as susceptible, less than 90% demonstrated resistance and between 90–97% was determined as resistance candidate (36, 39, 40).

## Results

**Fig. 2.**
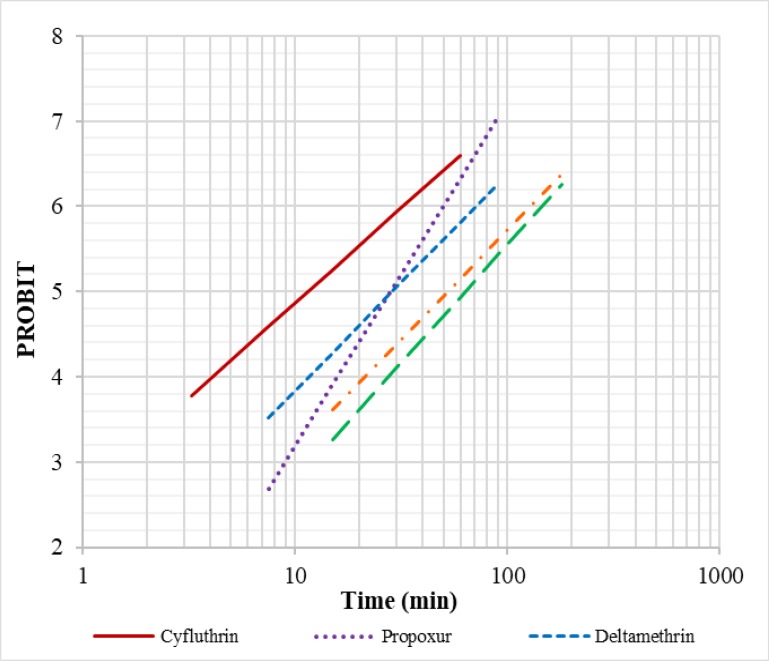
Regression lines of *Culiseta longiareolata* exposed to different group of insecticides in Marand County at East Azerbaijan Province, northwestern of Iran, 2017

Table 1 and 2 show the probit regression line parameters for females of *Cs. longiareolata* to different insecticides. In addition, Probit regression lines of insecticides against adult of *Cs. longiareolata* were drown which showed a linear relationship between mortality and time (Fig. 2).

**Table 1.** The Parameters of probit regression line of five insecticides on *Culisita longiareolata* in Marand County at East Azerbaijan Province, northwestern of Iran, 2017

**Insecticide Name**	**A**	**B±SE**	**LT_50_, (LCL-UCL) 95% C.I.**	**LT_90_, (LCL-UCL) 95% C.I.**	**X^2^ (df)**	**P value**
**Cyfluthrin**	−2.37	2.23±0.27	9.18	32.05	4.36 (3)	> 0.05
			11.53	43.37		
			14.27	68.03		

**DDT**	−4.41	2.57±0.32	43.35	125.78	5.8 (3)	> 0.05
			52.38	165.47		
			63.02	250.91		

**Deltamethrin**	−3.67	2.53±0.30	23.72	70.17	6.46 (3)	> 0.05
			28.79	92.24		
			34.8	138.22		

**Fenitrothion**	−5.01	2.78±0.34	53.26	140.51	2.39 (3)	> 0.05
			63.39	183.26		
			75.85	274.31		

**Propoxur**	−5.85	4.04±0.46	24.19	48.64	2.81 (3)	> 0.05
			28.05	58.2		
			32.36	74.68		

A= y-intercept, B= the slope of the line, SE= Standard error

LT_50_, 95% CI= Lethal Time causing 50% mortality and its 95% confidence interval

LT_90_, 95% CI= Lethal Time causing 90% mortality and its 95% confidence interval

LCL: Lower Confidence Limit, UCL: Upper Confidence Limit

*X*^2^= Heterogeneity about the regression line

df= degree of freedom, p= Represent heterogeneity in the population of tested

**Table 2.** Susceptibility level of *Culisita longiareolata* exposed to different groups of insecticides at diagnostic dose in Marand County at East Azerbaijan Province, northwestern of Iran, 2017

**Insecticides**	MR±EB^*^	Resistance status^**^
**Cyfluthrin**	95±0.25	RC
**DDT**	42.5±0.25	R
**Deltamethrin**	70±0.41	R
**Fenitrothion**	37.5±0.48	R
**Propoxur**	87.5±0.48	R

* Mortality Rate±Error Bar

** RC: Resistance Candidate

*** R: Resistance

**Fig. 3.**
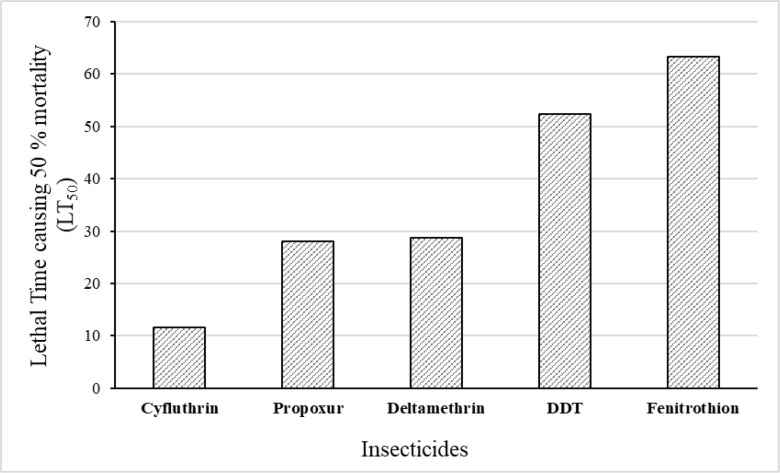
Lethal time causing 50% mortality of *Culiseta longiareolata* exposed to different group of insecticides in Marand County at East Azerbaijan Province, northwestern of Iran, 2017

The LT_50_ values were 52.38, 28.79, 11.53, 63.39 and 28.05min after treatment with DDT 4%, deltamethrin 0.05%, cyfluthrin 0.15%, fenitrothion 1% and propoxur 0.1%, respectively (Fig. 3). The highest toxicity against *Cs. longiareolata* was found on cyfluthrin (LT_50_: 11.53 and LT_90_: 43.37min) while the lowest toxicity was observed for fenitrothion 1% (LT_50_: 63.39 and LT_90_: 183.26min) (Table 1).

## Discussion

The excessive use of synthetic pesticides in agriculture plays an important role in the development of insecticide resistance in arthropods (41, 42). Resistance in medically important arthropods is developing and this is a major problem in their control (43).

Considering the current WHO criteria for insecticide resistance evaluation, *Cs. longiareolata* is resistant to Fenitrothion, DDT, deltamethrin, propoxur and candidate of resistance to cyfluthrin. Some studies showed that *Cs. longiaerolata* is resistance to DDT, propoxur, lambda-cyhalotrin and tolerant to malathion and deltamethrin more over LT_50_ value found as 131.94, 5.21, 17.60, 5.19 and 29.12min for DDT, deltamethrin, lambda-cyhalothrin, malathion and propoxur respectively (43). LT_90_ value of *Cs. longiaerolata* for DDT, deltamethrin, lambda-cyhalothrin, malathion and propoxur calculated as 588.13, 29.24, 229.26, 26.69 and 371.76 minutes respectively(41). Our results based on probit regression line showed that adult of *Cs. longiareolata* is more suseceptible to pyrethroid and carbamate insecticides. LT_50_ value of this species for DDT, cyfluthrin, deltamethrin, fenitrothion and propoxur calculated as 52.28, 11.53, 28.79, 63.39 and 28.05 minutes respectively. LT_90_ value found as 165.47, 43.37, 92.27, 183.26 and 58.2 minutes for DDT, cyfluthrin, deltamethrin, fenitrothion and propoxur respectively.

Previous studies reported that *Cs. Longiareolata* larvae was susceptible to *Bacillus sphaericus* and *B. thuringiensis* (44). Some reports showed that the LC_50_ and LC_90_ values of Novaluron (Insect Growth Regular) against *Cs. longiareolata* were reported as 0.51–0.91μg/l and 2.32–4.30μg/l, respectively (45).

In many regions of Iran, results of susceptibility test on *Cx. pipiens*, *Cx. quinquefasciatus*, *Anopheles stephensi*, and *Cs. longiareolata* showed that high resistant to different classes of insecticides, such as DDT, deltamethrin, lambda-cyhalothrin, propoxur and cyfluthrin and this finding is similar to our results for *Cs. longiareolata* (29–31, 41, 46, 47).

The lack of data on mosquito susceptibility to insecticides is a limiting factor for the success of control programs. Therefore, this finding can be useful in future vector control programs and investigations in order to prevent the development of resistance to insecticides.

Due to the emergence of resistance in mosquitoes to different classes of insecticides, the use of biological agents can be an effective method to control mosquitoes (42). However, the use of botanical insecticide, which have no adverse effects on the environment and humans, can be appropriate and an alternative control method for insecticide in vector control programs (48–55).

## Conclusion

This study confirms the resistance of the *Cs. longiareolata* to fenitrothion, DDT, deltamethrin, propoxur and candidate of resistance to cyfluthrin. When we observed the high resistance level of *Cs. longiareolata* increases to the insecticides in the study area, therefore, in order to avoid increasing resistance to insecticides, appropriate and effective strategies should be used such as: use of regular monitoring of current insecticides resistance, interventions in combination, rotations of insecticides, mixtures insecticides and plant insecticides. By using these appropriate methods and by decreasing the level of mosquitoes resistance to insecticides, it could be hopeful to better control the vector-borne diseases in the future.
